# Intraocular Infiltration

**DOI:** 10.4269/ajtmh.19-0539

**Published:** 2020-01

**Authors:** Koju Kamoi, Kyoko Ohno-Matsui

**Affiliations:** Department of Ophthalmology and Visual Science, Graduate School of Medical and Dental Sciences, Tokyo Medical and Dental University, Tokyo, Japan

A 58-year-old man presented with general malaise and showed positive results for human T-cell leukemia virus type 1 (HTLV-1) infection. As blood tests revealed abnormal leukocytes and flower cells, smoldering-type adult T-cell leukemia/lymphoma (ATL) was diagnosed. After 7 years, the condition changed to acute-type ATL. Blood tests identified an increase in CD4 from 995 cells/µL to 1,256 cells/µL and monoclonal integration of HTLV-1 provirus into tumor cells. Treatment was started with chemotherapy and allogeneic bone marrow transplantation. During follow-up, he was referred to the ophthalmology department after experiencing sudden visual loss in the right eye.

**Figure 1.**
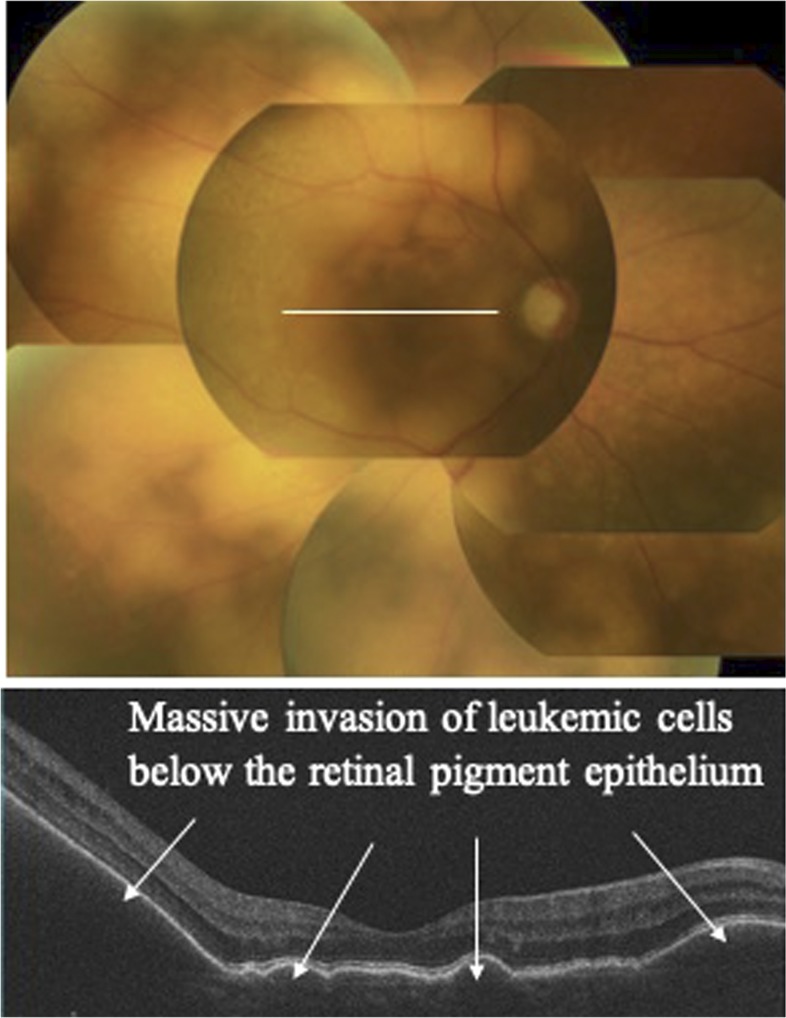
Yellowish-white infiltrative foci associated with protrusions in the retina (upper figure). Optical coherence tomography reveals massive solid infiltrative foci below the retinal pigment epithelium (lower figure). White line represents scan line. This figure appears in color at www.ajtmh.org.

**Figure 2.**
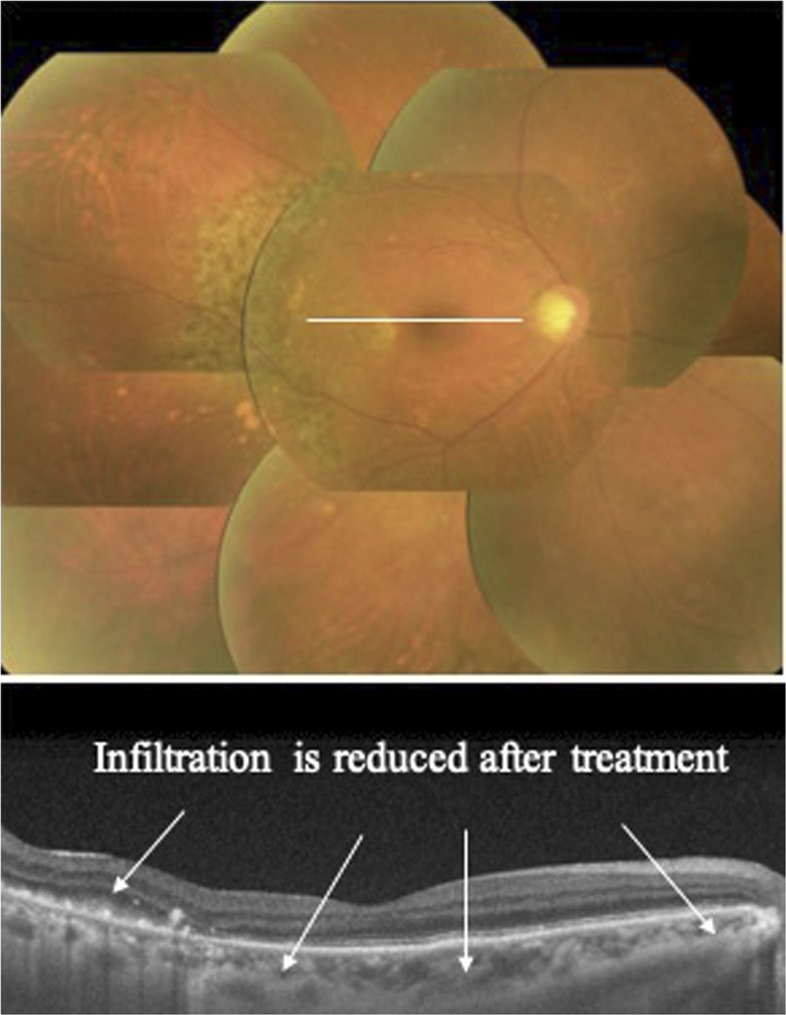
Infiltration is reduced 6 months after vitreous injection of methotrexate concomitant with radiotherapy. This figure appears in color at www.ajtmh.org.

Slit-lamp examination revealed cellular infiltration in the anterior chamber and vitreous humor. Fundus examination showed yellowish-white infiltrative foci associated with protrusions in the retina. Optical coherence tomography revealed massive solid infiltrative foci below the retinal pigment epithelium (Figure 1). As differential diagnoses for co-infections of the eye, our recent nationwide survey revealed cytomegalovirus (CMV) as a major cause of opportunistic infection in the eye of ATL patients, followed by herpesvirus and toxoplasma.^1^ In addition, co-infections of the eye in immunocompetent patients are not frequently confirmed using serologic diagnostic methods.^2^ Multiplex polymerase chain reaction (PCR) and broad-range PCR of a sample of aqueous humor, therefore, ruled out viral (human simplex virus-1 and 2, varicella zoster virus, Epstein–Barr virus, CMV, and human herpesvirus 6–8), toxoplasma, tuberculosis, syphilis, and bacterial and fungal infections, ruling out opportunistic infection and allowing the diagnosis of intraocular leukemic cell infiltration.^3^ Elevated lesions on the retina improved after two vitreous injections of 400 μg/0.1 mL of methotrexate and subsided with five sessions of 2-Gy radiotherapy. Corresponding to this combination therapy, retinal lesions gradually resolved within 6 months (Figure 2).

Globally, HTLV-1 infection is now the focus of attention, since the discovery that more than 40% of adults from Aboriginal communities in central Australia are infected with HTLV-1.^4^ This infection causes ATL and also visual impairment by ATL-related ocular manifestations.^5^ As HTLV-1–associated ATL is a neoplasia characterized by massive invasion of leukemic cells into various organs, physicians should keep in mind that intraocular infiltration is the most frequent manifestation observed among HTLV-1–associated ATL patients.^1^
